# Exploration of the active components and mechanisms of Epimedium for the treatment of Undescended Testis

**DOI:** 10.3389/fendo.2025.1642197

**Published:** 2025-08-27

**Authors:** Zhonglei Deng, Sirui Zhang, Haiyang Zhao, Shijia Xu, Miao Qi, Tianle Pu, Hui Wang

**Affiliations:** ^1^ Department of Urology, The Second Affiliated Hospital of Nanjing Medical University, Nanjing, China; ^2^ State Key Laboratory of Reproductive Medicine and Offspring Health, Department of Histology and Embryology, School of Basic Medical Sciences, Nanjing Medical University, Nanjing, China; ^3^ Chinese Medicine Research Institute, Engineering Research Center of Modern Preparation Technology of Traditional Chinese Medicine, Ministry of Education, Shanghai University of Traditional Chinese Medicine, Shanghai, China; ^4^ The First School of Clinical Medicine, Nanjing Medical University, Nanjing, China; ^5^ The Second School of Clinical Medicine, Nanjing Medical University, Nanjing, China

**Keywords:** Undescended Testis, Epimedium, network pharmacology, Mendelian randomization, Traditional Chinese Medicine

## Abstract

**Background:**

Undescended Testis, the most common congenital male reproductive disorder in children, demonstrates complex pathogenesis with genetic predisposition. While early surgical intervention (before 18 months) remains standard care, pharmacological options are limited.

**Purpose:**

Explore the therapeutic potential of Traditional Chinese Medicine for Undescended Testis.

**Study design:**

This study employs multiple bioinformatics approaches to search for potential Traditional Chinese Medicines for Undescended Testis treatment and investigate their therapeutic mechanisms.

**Methods:**

This study integrated medication regularity analysis, network pharmacology, Mendelian randomization, and molecular docking, with exploration conducted through cellular and animal models.

**Results:**

Our analysis identified 27 bioactive Epimedium compounds, with Quercetin, Kaempferol, and Icariin showing particular promise. Protein interaction networks revealed AKT1, PARP1, and PGR as key targets. Functional enrichment analysis primarily focused on biological processes including response to stress-induced damage (such as reactive oxygen species, chemical toxins, and other stressors), steroid signaling and receptor activation, transmembrane signal transduction, as well as cellular proliferation and apoptosis regulation. Molecular docking demonstrated Yinyanghuo B’s strong binding affinity with PARP1 (-7.2 kcal/mol). Mendelian randomization confirmed PARP1 reduction’s causal relationship with lower Undescended Testis risk. We constructed animal and cellular heat stress models to simulate Undescended Testis, and subsequent experiments observed significant DNA damage. Thermally stressed TM4 cells showed significant PARP1 upregulation, suggesting its critical role in testicular heat stress response by accelerating DNA damage repair.

**Conclusion:**

These findings potentially provide mechanistic evidence supporting Epimedium’s traditional use while advancing Traditional Chinese Medicine modernization through contemporary pharmacological validation.

## 1 Introduction

Undescended Testis is a common congenital disorder in which the testes fail to descend into the scrotum at birth, affecting the reproductive health of male infants (1). Epidemiological studies have shown that the incidence of congenital Undescended Testis ranges from 1% to 9% (2, 3). The condition demonstrates progressive histopathological deterioration: interstitial fibrosis emerges by age 1, followed by germ cell depletion and peritubular fibrosis at age 2, with subsequent mitochondrial degeneration and collagen accumulation in older children (4). Beyond impaired spermatogenesis (manifesting as oligoasthenoteratospermia), long-term sequelae include reduced fertility, increased risk of testicular cancer, and psychological impacts due to appearance (5).

Sustained exposure of the testes to suprascrotal temperatures is widely recognized as a key pathological mechanism in Undescended Testis, inducing chronic heat stress (6). Research indicates that this persistent chronic heat stress impairs male reproductive health by disrupting the spermatogenic microenvironment. Key mechanisms include damage to Sertoli cell structure and function and compromised integrity of the blood-testis barrier (7). In experimental research, the acute heat stress model is widely used to study early testicular heat stress responses, as it shares some core mechanisms with chronic heat stress (8).

The etiology of Undescended Testis involves multifactorial interactions, with emerging evidence highlighting environmental influences alongside hormonal and genetic factors (9). Surgical intervention via orchiopexy before 18 months remains the gold standard, preventing testicular dysgenesis and preserving fertility (10). However, surgical limitations persist for complex cases, accompanied by inherent procedural risks. Endocrine therapies using human chorionic gonadotropin or gonadotropin-releasing hormone (hCG/GnRH) demonstrate efficacy in boosting germ cell counts (11, 12), yet histopathological analyses reveal transient adverse effects. These include elevated intratesticular pressure, testicular congestion, and germ cell apoptosis (13). Therefore, it is crucial to seek more effective and safer pharmacological treatments or adjuvant strategies.

Traditional Chinese Medicine (TCM) posits that the fundamental pathogenesis of male infertility is the deficiency of kidney essence, with the kidney being the “foundation of reproduction,” directly influencing reproductive function (14). Traditional Chinese Medicine offers novel therapeutic perspectives through its holistic approach. For example, Semen Cuscutae-Fructus Lycii ameliorates spermatogenic dysfunction by suppressing oxidative stress-induced ferroptosis through the Nrf2/HO-1 signaling pathway (15); Ginsenoside Rg1 upregulates expression levels of Cx43/p-PI3K/mTOR, mitigating testicular aging (16); Icariin, derived from Epimedium, enhances testosterone biosynthesis by upregulating steroidogenic enzyme activity and ameliorates testicular dysfunction in mice (17); *In vitro* studies have also found that Icariin can promote the proliferation of Sertoli cells and improve the spermatogenic microenvironment by regulating the ERK1/2 pathway (18). The spermatogenic microenvironment, particularly Sertoli cells, plays a critical role in both the development and prevention of Undescended Testis, making the identification of protective components essential. Leydig cells are also essential for testicular descent via secretion of testosterone and insulin-like peptide 3(INSL3). INSL3 promotes gubernacular swelling and cord shortening, while testosterone drives the inguinoscrotal phase (19, 20). In cryptorchidism, oxidative stress impairs Leydig cell function, reducing testosterone. Rubus apetalus enhances reproductive function by boosting testosterone synthesis (21). While traditional Chinese herbs such as Epimedium, licorice, and Poria cocos show potential as therapeutic options, their specific molecular targets and regulatory pathways remain undefined.

Contemporary methodologies enable systematic exploration of TCM mechanisms: Medication regularity analysis is a process of systematically sorting out and mining drug use data (such as prescription, drug use record, therapeutic effect feedback, etc.) to find the common characteristics and potential laws of drug use. Network pharmacology identifies polypharmacological targets through protein interaction mapping. Mendelian randomization establishes causal relationships using genetic instrumental variables. Molecular docking predicts ligand-receptor binding affinities with <2Å resolution. *In vitro* and *in vivo* models validate the theoretical results.

This study aims to identify key TCMs for treating Undescended Testis through medication regularity analysis and network pharmacology, exploring their material basis to offer new insights and theoretical evidence. By integrating methods like Mendelian randomization, molecular docking, and in cell and animal experiments, we seek to elucidate potential mechanisms and provide feasible treatment suggestions, advancing the modern and precise application of TCM.

## 2 Materials and methods

### 2.1 Medication regularity analysis of Undescended Testis

Using databases such as CNKI, VIP, Wanfang Data, Web of Science, Elsevier ScienceDirect, SpringerLink, and NCBI, we initially used the search expression [“Undescended Testis” AND “medication regularity analysis”] and found no relevant articles. We then modified the search expression to [“oligospermia” AND “medication regularity analysis”] OR [“asthenospermia” AND “medication regularity analysis”] OR [“teratospermia” AND “medication regularity analysis”]. A total of 18 relevant articles were included, and the frequency of drug use reported in these studies were statistically analyzed. The top twenty drugs by usage frequency are shown in **Table 1**.

**Table 1:** Medication regularity analysis of sperm disorders (OAT).

No.	Drug	Frequency(times)
1	Semen cuscuta	3242
2	Astragalus	2468
3	Epimedium	2418
4	Goji berry	2322
5	Angelica sinensis	2135
6	Rubus idaeus	1629
7	Schisandra chinensis	1582
8	Licorice	1426
9	Achyranthes bidentata	1339
10	Plantago asiatica	1282
11	Morinda officinalis	1241
12	Poria cocos	1212
13	Rehmannia glutinosa	1158
14	Cistanche deserticola	1088
15	Curculigo orchioides	1062

OAT, Oligospermia, Asthenospermia, Teratospermia.

### 2.2 Network pharmacology analysis

#### 2.2.1 Identification of active components and targets of Epimedium

We searched the TCMSP (https://old.tcmsp-e.com/tcmsp.php) database for the chemical components of Epimedium, setting the screening criteria as oral bioavailability (OB) ≥ 30% and drug-likeness (DL) ≥ 0.18, and combined with the 2020 edition of the Chinese Pharmacopoeia to manually screen out the active components of Epimedium (Icariin, Epimedin A, Epimedin B and Epimedin C), summarizing the effective components of Epimedium. We used the TCMSP and SwissTargetPrediction (http://swisstargetprediction.ch/) databases to search for and predict the targets of the active components, and corrected the gene names of the active component targets using the UniProt database, removing duplicate targets.

#### 2.2.2 Identification of targets related to Undescended Testis

Using the Genecards (https://www.genecards.org/) database, we searched for targets related to Undescended Testis, removing duplicates. We used the R package “*VennDiagram*” to obtain the intersection targets of the active components of Epimedium and Undescended Testis, and drew a Venn diagram.

#### 2.2.3 Construction of Protein-Protein Interaction network and screening of key targets

We performed PPI analysis on the intersection targets obtained in 2.2 using the STRING (https://cn.string-db.org/) database, with the species set to “Homo sapiens” and the minimum interaction score set to 0.4, hiding unrelated proteins.

#### 2.2.4 Gene Ontology and Kyoto Encyclopedia of Genes and Genomes pathway enrichment analysis

Using the R packages “*enrichplot*” and “*ggplot2*”, we performed GO and KEGG functional enrichment analysis on the intersecting targets. GO enrichment analysis included biological processes (BP), cellular components (CC), and molecular functions (MF). We selected the top 10 items according to ascending P-values (i.e., most significant) and drew charts for GO and KEGG.

### 2.3 Mendelian randomization analysis

#### 2.3.1 Tool variable acquisition and screening

The intersection targets of Epimedium and Undescended Testis were used as exposure variables. We used cis-pQTL summary data from the deCODE (22) and UK Biobank Pharma Proteomics Project (UKB-PPP) (https://registry.opendata.aws/ukbppp/) projects as exposure variables, screening with criteria of *P*<1e-6, r^2^<0.01, and clump processing with kb=5000. The outcome variable (Undescended Testis) was derived from the FinnGen Documentation of R12 release.

#### 2.3.2 Mendelian randomization analysis and secondary validation

We performed two-sample Mendelian randomization (MR) analysis based on the R package “*TwoSampleMR*”. We assessed the causal relationship between the intersection targets and Undescended Testis using causal effect estimation methods including inverse variance weighted (IVW), MR-Egger regression, simple mode, weighted median, and weighted mode. *P*<0.05 was considered statistically significant.

For targets with significant causal relationships with Undescended Testis (*P*<0.05), we performed secondary validation. Using publicly available data from GTEx v8 (https://www.gtexportal.org/), we obtained cis-eQTLs related to the targets across 49 human tissues and used these cis-eQTLs as instrumental variables for two-sample MR analysis. The assessment methods included inverse variance weighted (IVW), MR-Egger regression, simple mode, weighted median, and weighted mode. Odds ratios (OR) and their 95% confidence intervals (95%CIs) were estimated, and a *p*-value < 0.05 was considered statistically significant.

### 2.4 Molecular docking

We conducted molecular docking to analyze causal targets from MR analysis and active components of Epimedium. Molecular docking predicts protein-ligand binding modes and interaction strengths by identifying optimal conformations with minimal binding free energy.

Protein Preparation: 3D protein structures from the PDB database were imported into AutoDock4. Water molecules were removed, hydrogen atoms were added, and files were converted to PDBQT format.

Ligand Preparation: Compound 3D structures from PubChem were similarly processed in AutoDock4, with torsion trees configured automatically before exporting to PDBQT format.

Docking & Visualization: Receptor and ligand PDBQT files were docked in AutoDock4 using a protein-centered grid box to ensure full coverage. The lowest-energy conformation was selected and visualized using PyMol 2.5.

### 2.5 *In vitro* model validation

#### 2.5.1 Cell culture

Normal mouse testicular Sertoli cells (TM4, CL-0456) and complete medium (CM-0456) were purchased from Nanjing Wingfleas Biotechnology Co., Ltd. (Nanjing, China). Under conventional conditions, cells were cultured at 37°C with 5% CO_2_.

#### 2.5.2 Model construction

Cells without any treatment were used as the blank group; TM4 cells with 80% confluence were treated with 42°C warm water bath for 20 minutes to simulate the heat stress environment of Undescended Testis, serving as the Undescended Testis model group (8, 23, 24).

#### 2.5.3 qPCR

RNA was extracted using an RNA extraction kit (G3640) purchased from Wuhan Seaweed Biotechnology Co., Ltd. (Wuhan, China); RNA was reverse-transcribed into cDNA using a reverse transcription kit (R223-01) purchased from Nanjing Novozyme Biotechnology Co., Ltd. (Nanjing, China); SYBR Green Premix (11201ES08) was added to the amplification system along with the template, primers, and SYBR Green Premix for real-time quantitative PCR to measure the mRNA expression levels of GAPDH, STAT1, RASA1, PGR, IGFBP3, PARP1, and CALCA. The SYBR Green Premix was purchased from Yeasen Biotechnology Co., Ltd. The qPCR instrument (A28575) was purchased from Thermo Fisher Scientific (MA, USA).

RNA was extracted using an RNA extraction kit (G3640, Wuhan Seaweed Biotechnology). cDNA was synthesized from RNA using a reverse transcription kit (R223-01, Nanjing Novozyme Biotechnology). For qPCR, the amplification system included SYBR Green Premix (11201ES08, Yeasen Biotechnology), template, and primers to measure mRNA expression of…

The primers used in this experiment were as follows:

GAPDH:
5’-TCGGATCAACGGATTTGGT-3’ (forward);
5’-TTCCCGTTCTCAGCCTTGAC-3’ (reverse);
STAT1:
5’- GCTGCCTATGATGTCTCGTTT-3’ (forward);
5’- TGCTTTTCCGTATGTTGTGCT-3’ (reverse);
RASA1:
5’- GGCCCTCAGATAATACTCCTGG-3’ (forward);
5’- GCGGCCTCCCATCATAAACT-3’ (reverse);
PGR:
5’- CCAGACGGAAAGACAGGGG-3’ (forward);
5’- CCTTCCCTATGAGTGGCTTCT-3’ (reverse);
IGFBP3:
5’- TCTAAGCGGGAGACAGAATACG-3’ (forward);
5’- CTCTGGGACTCAGCACATTGA-3’ (reverse);
PARP1:
5’- GCTTTATCGAGTGGAGTACGC-3’ (forward);
5’- GGAGGGAGTCCTTGGGAATAC-3’ (reverse);
CALCA:
5’- GAGGGCTCTAGCTTGGACAG-3’ (forward);
5’- AAGGTGTGAAACTTGTTGAGGT-3’ (reverse).

The primers were purchased from GenScript Biotechnology Co., Ltd. (NJ, USA).

#### 2.5.4 TUNEL staining

Cells grown on coverslips were washed three times with PBS and fixed with pre-chilled immunostaining fixative for 20 minutes. After three additional washes with PBS, the cells were permeabilized with 0.1% Triton X-100 for 5 minutes. Then, 100 µL of 1× Equilibration Buffer was added and equilibrated at 25°C for 20 minutes, followed by the addition of 50 µL of TdT and incubation at 25°C for 60 minutes. The cells were mounted with DAPI and observed under a fluorescence microscope after 2 hours. The TUNEL assay kit (A113) was purchased from Nanjing Novizan Biotechnology Co., Ltd.

#### 2.5.5 Western blot

Treated cells were collected and lysed in 1× RIPA lysis buffer (P0013C) containing protease inhibitors to extract proteins. Protein concentration was determined using the BCA method (P0011). A total of 30 µg of protein samples were separated by 10% SDS-PAGE gel and transferred onto nitrocellulose membranes. The membranes were blocked with 5% skim milk at room temperature for 1 hour, followed by incubation with specific primary antibodies at 4°C overnight: HSP70 (66183-1-Ig) and GAPDH (60004-1-Ig). Subsequently, the membranes were incubated with horseradish peroxidase-conjugated secondary antibodies (A0208) at room temperature for 1 hour. Target proteins were detected using ECL luminescent solution (FD8000), and immunoblot images were acquired and analyzed. The lysis buffer, BCA kit, and secondary antibodies were purchased from Beyotime Biotechnology (Shanghai, China); HSP70 and GAPDH antibodies were purchased from Wuhan Sanying Biotechnology (Wuhan, China).

### 2.6 *In vivo* model validation

#### 2.6.1 Mouse feeding

All mice were raised in a specific pathogen-free environment. All animal protocols were approved by the Animal Care and Use Committee of Nanjing Medical University (IACUC-2402036). All mice were purchased from Jiangsu GemPharmatech Co., Ltd. (Nanjing, China). The animals were randomly divided into groups, with 5 mice per group.

#### 2.6.2 Model construction

Nine-week-old male C57B6 wild-type mice were selected. Mice without any treatment served as the blank control group, while mice with testes subjected to a 42°C warm water bath for 20 minutes served as the model group.

#### 2.6.3 Histopathological analysis

After fixation with 4% paraformaldehyde for 48 h, testicular tissues were subjected to gradient alcohol dehydration and paraffin embedding for histopathological evaluation. Subsequently, 5-μm sections were obtained and mounted on slides, followed by drying for subsequent usage. H&E staining (G1001, Servicebio) was employed to visualize the tissue sections. The vacuolation rate of seminiferous tubules was quantified and statistically analyzed using ImageJ software. Specifically, five non-consecutive sections were randomly selected from each mouse, and the number of seminiferous tubules with vacuolation was counted in each section. The number of total tubules and those showing vacuolation (including tubules containing only Sertoli cells or evident vacuoles) were counted. The vacuolation rate was calculated using the formula:(Number of Sertoli cell-only tubules + vacuolated tubules)/Total tubules*100%. The data were subjected to statistical analysis and presented in the form of a bar graph for further analysis.

#### 2.6.4 TUNEL staining

Testes from treated mice were collected, fixed, and embedded in paraffin for sectioning. Paraffin sections were subjected to TUNEL staining. The sections were dewaxed in a 65°C oven for 2 hours, immersed in xylene twice at 37°C for 15 minutes each, and then incubated in absolute ethanol twice for 5 minutes each, followed by incubation in 90%, 80%, and 70% ethanol for 5 minutes each. The sections were rinsed with ddH_2_O for 10 minutes. They were then fixed with pre-chilled immunostaining fixative for 20 minutes. After washing three times, the sections were permeabilized with 0.1% Triton X-100 for 5 minutes. Next, 100 µL of 1×Equilibration Buffer was added and equilibrated at 25°C for 20 minutes, followed by the addition of 50 µL of TdT and incubation at 25°C for 60 minutes. The sections were mounted with DAPI and observed under a fluorescence microscope after 2 hours.

## 3 Results

**Figure 1:**
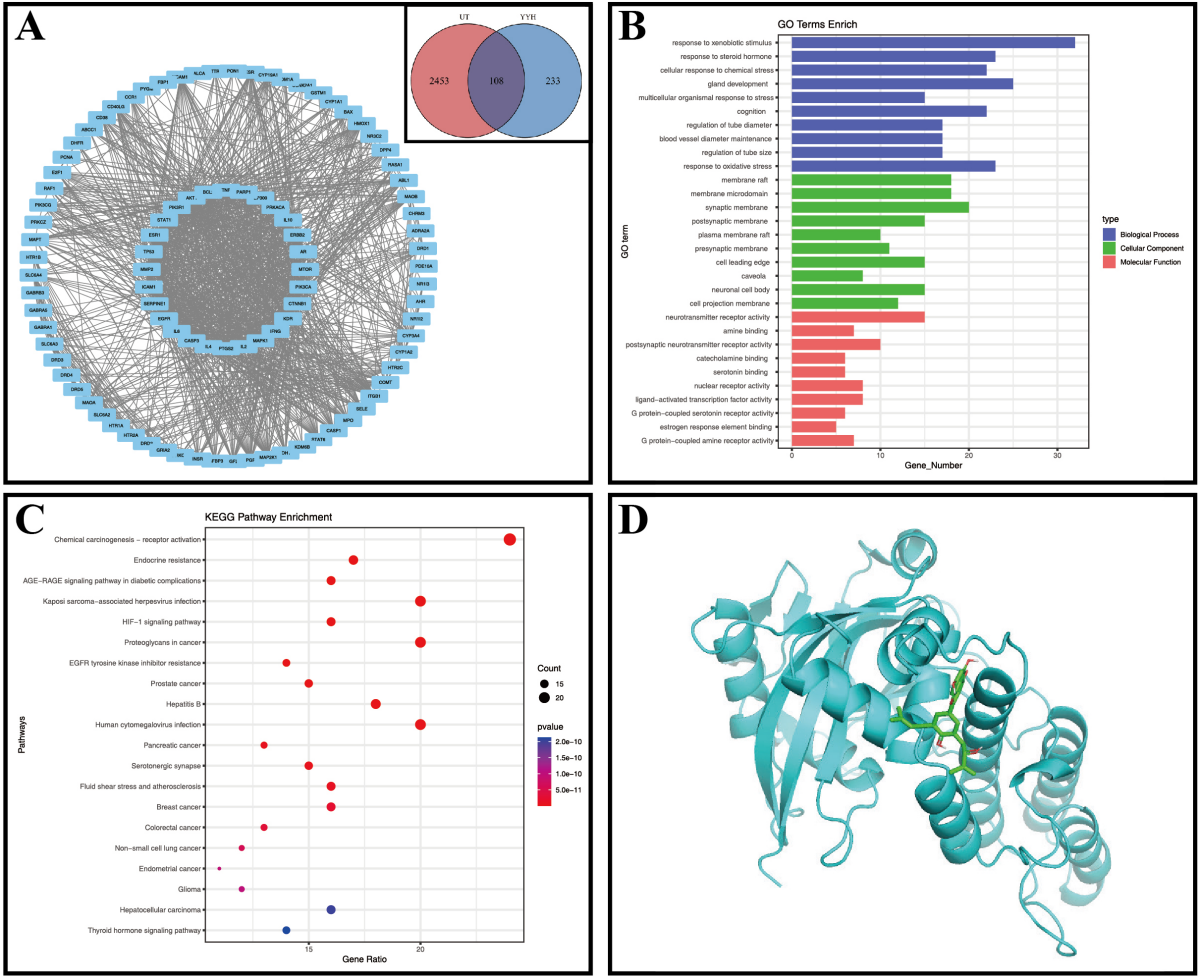
Network pharmacology figures. **(A)** Venn diagram, UT, Undescended Testis, YYH, Epimedium; PPI network diagram; **(B)** GO enrichment analysis; **(C)** Bubble chart of KEGG enrichment analysis; **(D)** Molecular docking diagram of Yinyanghuo B and PARP1.

Our study identified Epimedium as a key herb among Traditional Chinese Medicines used for treating sperm-related diseases, with 27 active components including Quercetin, Kaempferol, Icariin, and Yinyanghuo B (**Table 2**). Through network pharmacology analysis, we discovered 341 potential targets for Epimedium, 108 of which overlapped with 2561 known Undescended Testis-related targets (**Figure 1A**). Protein-protein interaction analysis (**Figure 1A**) revealed critical nodes such as AKT1, TP53, MAPK1, PRKACA, PARP1, and CTNNB1, while GO enrichment (**Figure 1B**) highlighted biological processes including oxidative stress response and tube diameter regulation. KEGG pathway analysis (**Figure 1C**) implicated key signaling mechanisms like the HIF-1 pathway and endocrine resistance.

**Table 2:** Effective components of Epimedium.

No.	Component name	PubChem ID
1	Quercetin	5280343
2	Kaempferol	5280863
3	Icaritin	5318980
4	Icariside II	5488822
5	Luteolin	5280445
6	Yinyanghuo B	5315394
7	Icariside I	5745470
8	C-Homoerythrinan, 1,6-didehydro-3,15,16-trimethoxy-, (3.beta.)-	/
9	8-(3-methylbut-2-enyl)-2-phenyl-chromone	17861868
10	8-Isopentenyl-kaempferol	129716399
11	Anhydroicaritin	14583584
12	DFV	5273570
13	Chryseriol	5280666
14	1,2-bis(4-hydroxy-3-methoxyphenyl)propan-1,3-diol	12468616
15	Magnograndiolide	5319198
16	Yinyanghuo A	5315393
17	Sitosterol	12303645
18	6-hydroxy-11,12-dimethoxy-2,2-dimethyl-1,8-dioxo-2,3,4,8-tetrahydro-1H-isochromeno[3,4-h]isoquinolin-2-ium	12115137
19	Yinyanghuo E	5315397
20	Yinyanghuo C	5315395
21	Linoleyl acetate	5319042
22	Olivil	5273570
23	Icariside A7	5318401
24	Poriferast-5-en-3beta-ol	457801
25	24-epicampesterol	5283637
26	Anhydroicaritin-3-O-alpha-L-rhamnoside	/
27	Icariin	5318997

**Figure 2:**
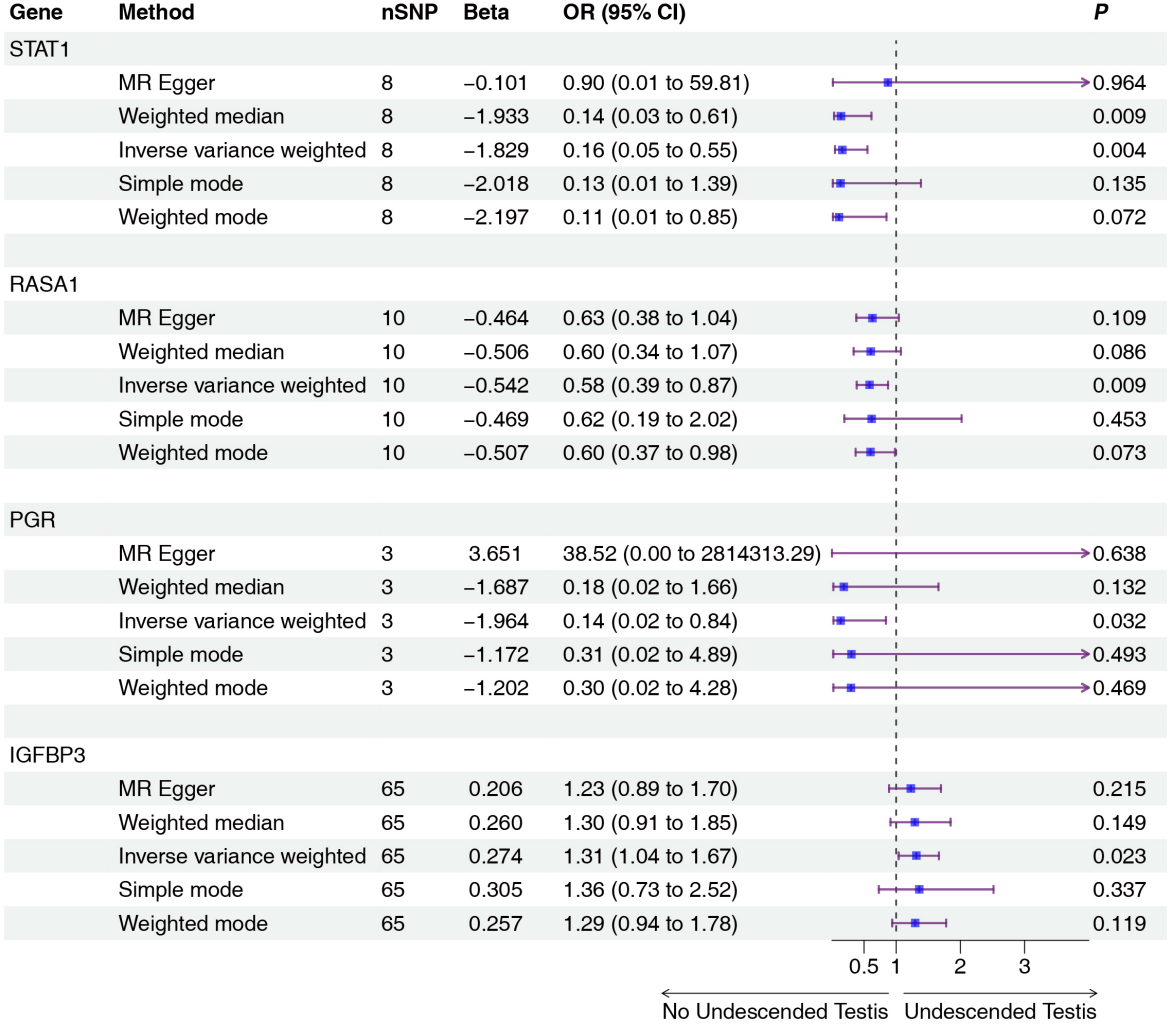
Two-sample MR analysis of intersection targets pQTL (deCODE) and Undescended Testis. Gene, Target gene; nSNP, Number of effective SNPs (*P*<1e-6); OR, Odds Ratio; 95% CI, 95% Confidence Interval; *P*, p-value corresponding to SNPs in MR results.

**Figure 3:**
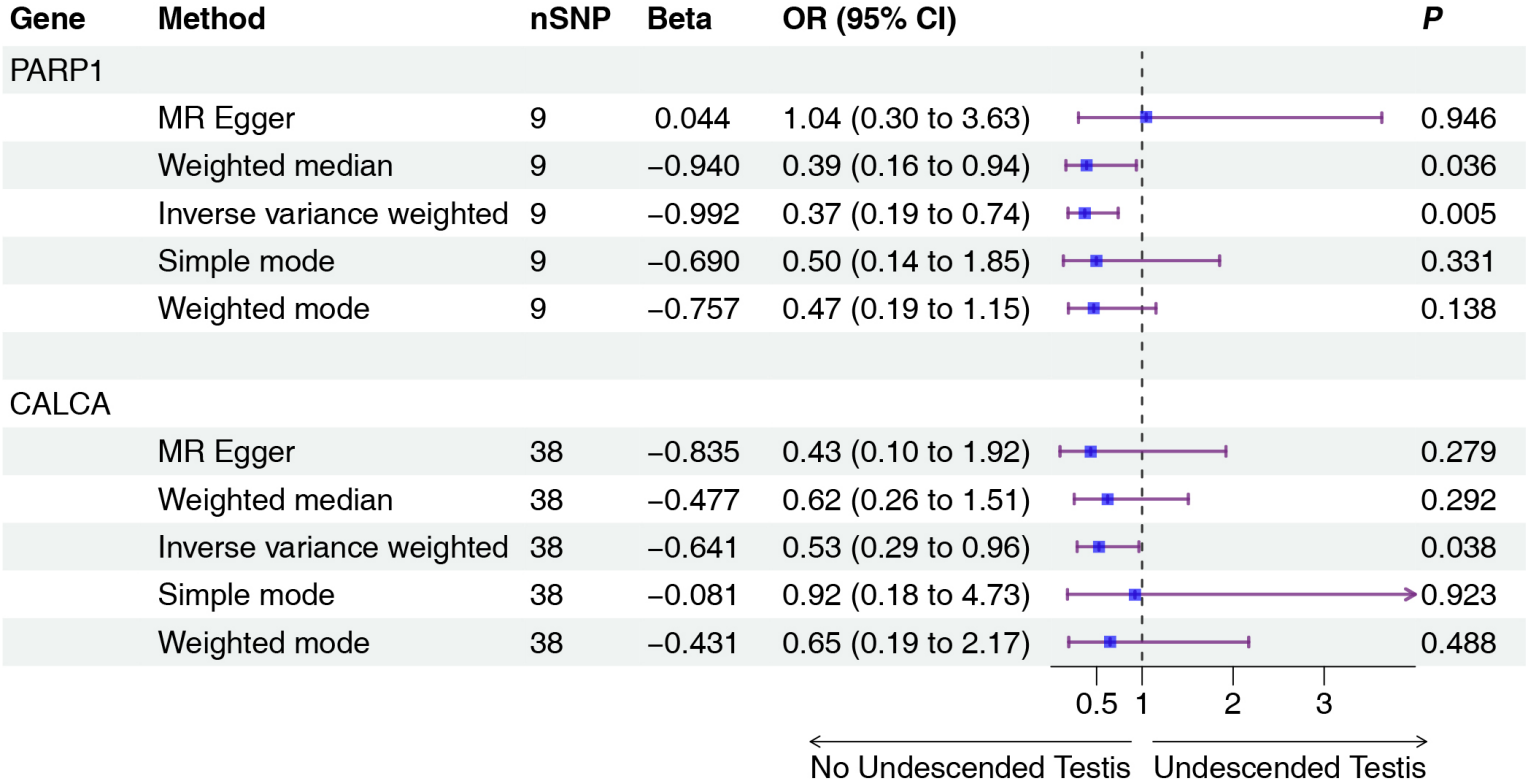
Two-sample MR analysis of intersection targets pQTL (UKB-PPP) and Undescended Testis. Gene, Target gene; nSNP, Number of effective SNPs (*P*<1e-6); OR, Odds Ratio; 95% CI, 95% Confidence Interval; *P*, p-value corresponding to SNPs in MR results.

**Figure 4:**
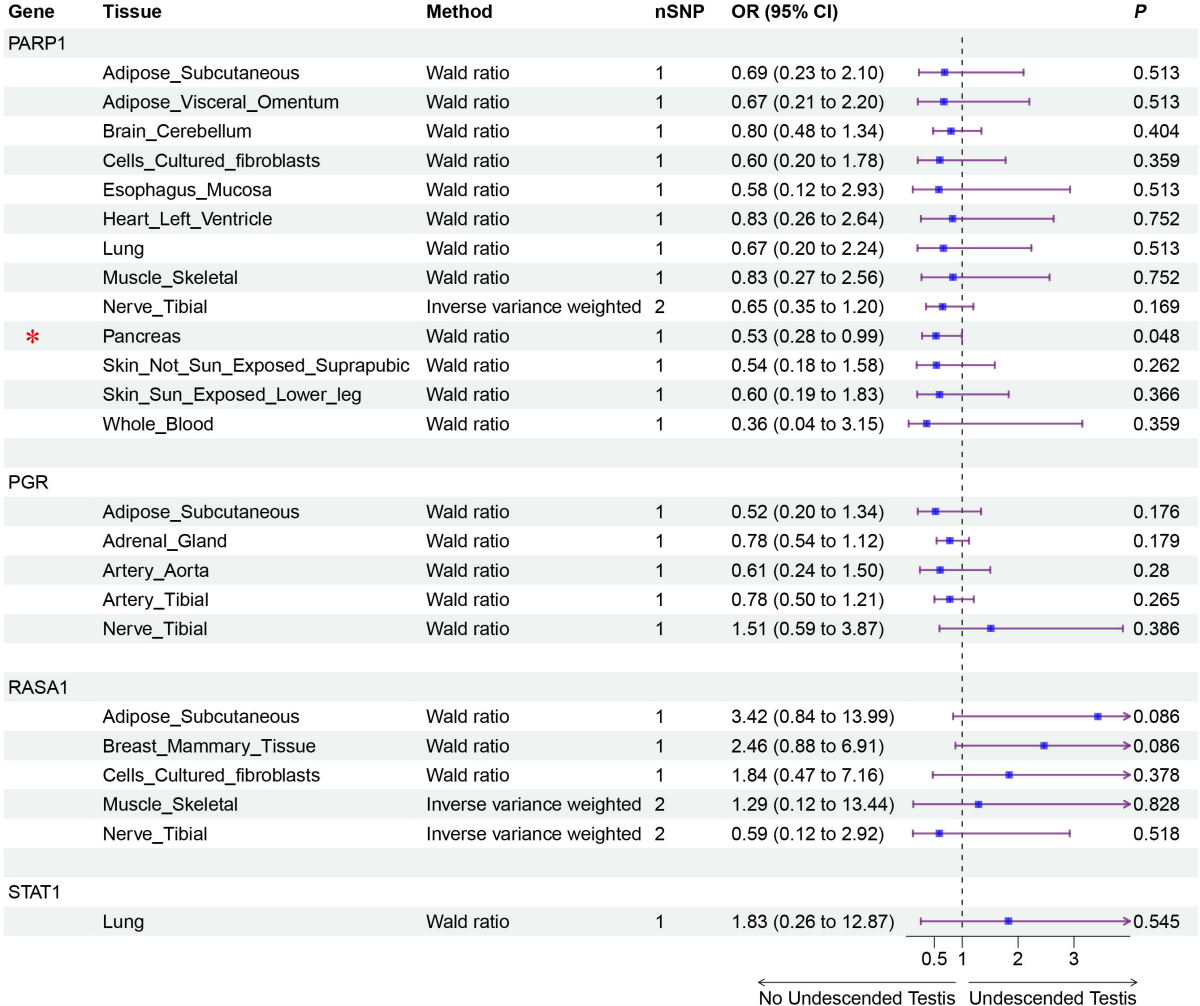
Validation MR analysis of positive genes eQTL and Undescended Testis. Gene, Target gene; Tissue, Tissue where SNPs are located; nSNP, Number of effective SNPs (*P*<1e-6); OR, Odds Ratio; 95% CI, 95% Confidence Interval; *P*, p-value corresponding to SNPs in MR results; an asterisk “*” denotes *P*<0.05 as a statistically significant result in the Mendelian randomization analysis.

Mendelian randomization analysis using pQTL data identified six proteins with significant associations (**Figures 2**, **3**): IGFBP3 increased disease risk (OR=1.31, 95% CI: 1.04-1.67, P=0.023), while STAT1 (OR=0.16, 95% CI: 0.05-0.55, P=0.004), RASA1 (OR=0.58, 95% CI: 0.39-0.87, P=0.009), PGR (OR=0.14, 95% CI: 0.02-0.84, P=0.032), PARP1 (OR=0.37, 95% CI: 0.19-0.74, P=0.005), and CALCA (OR=0.53, 95% CI: 0.29-0.96, P=0.038) showed protective effects. Subsequent eQTL (**Figure 4**) analysis confirmed PARP1’s causal relationship in pancreatic tissue (OR=0.53, 95% CI: 0.28-0.99, P=0.048).

**Figure 5:**
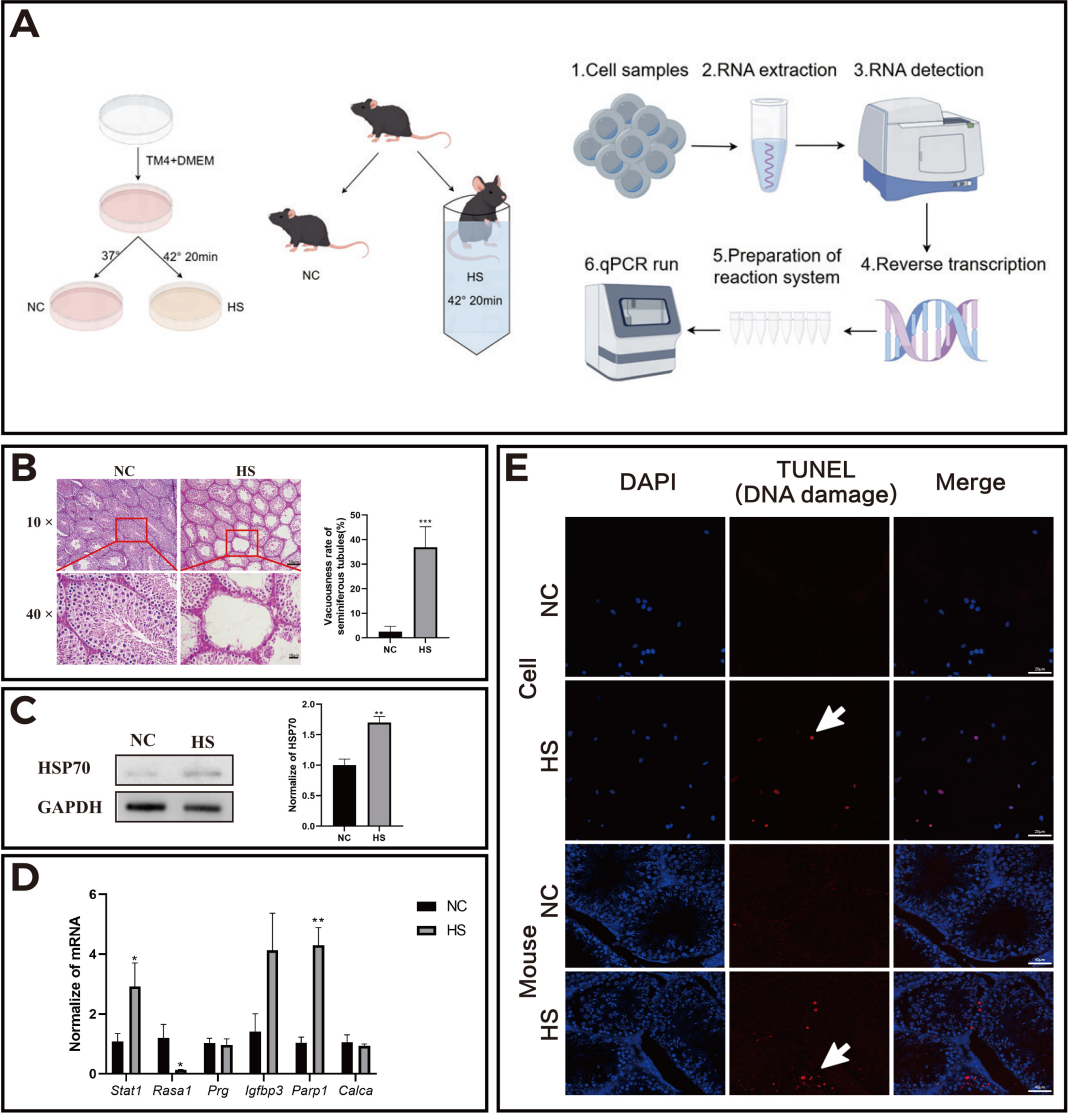
HS increased the apoptosis level of mouse testicular cells and changed the transcriptional activity of related genes. **(A)** Schematic representation of the *in vitro* (TM4 cell line) and *in vivo* (mouse) experimental models; **(B)** Results of H&E staining of mouse testes in each group; **(C)** HSP70 protein expression in the normal control (NC) and heat stress (HS) groups; **(D)** Transcriptional activity of related genes in the NC and HS groups; **(E)** Apoptosis levels in TM4 cells and testicular tissue sections of mice. *P<0.05, **P<0.01, compared with control.

Experiments validated heat stress (HS)-induced reproductive system damage using TM4 cells and mouse models (**Figure 5A**). The results of H&E staining in histology showed that there were obvious cavities in the seminiferous tubules in the heat stress group (HS) compared to the control group, with a significant decrease in the number of cells in the tubules. The seminiferous tubules with disrupted structure, obviously irregular arrangement, and sharply decreased Sertoli cells, presenting vacuolar changes were counted as vacuolar tubule numbers. The ratio of vacuolar tubule numbers to total tubule numbers was calculated (**Figure 5B**). Western blot analysis confirmed elevated HSP70 protein levels following HS treatment, demonstrating successful HS modeling (**Figure 5C**). qPCR analysis showed upregulated transcriptional activity of Stat1 and Parp1, and downregulated expression of Rasa1 (**Figure 5D**), aligning with bioinformatics predictions. TUNEL assays demonstrated significantly increased DNA damage in HS-treated TM4 cells and testicular tissues compared to the controls (**Figure 5E**), confirming HS-triggered DNA damage and apoptosis.

We performed molecular docking between PARP1 and its corresponding Epimedium component, Yinyanghuo B, and the results showed a minimum binding energy of -7.2 kcal/mol (less than -7 kcal/mol), indicating a stable binding between PARP1 and Yinyanghuo B (**Figure 1D**).

## 4 Discussion

Undescended Testis, a complex congenital male reproductive disorder, presents limited therapeutic options and frequent complications, highlighting the need for novel adjunct treatments. Traditional Chinese Medicine has long utilized Epimedium for reproductive conditions. Animal studies (25–27) demonstrate that its active components -Icariin and total flavonoids- alleviate testicular damage through anti-inflammatory and antioxidant mechanisms. Compounds found in Epimedium, such as Epimedium mixtures and β-sitosterol, enhance male reproductive function by modulating androgen levels, while Icariin, Quercetin, Kaempferol, and Luteolin improve the spermatogenic microenvironment. Total flavonoids and Icariin II (ICAII) promote spermatogenesis itself (24, 28, 29). Specifically, Icariin helps maintain blood-testis barrier (BTB) integrity by inhibiting the p38 MAPK/MMP9 signaling pathway, thereby increasing the expression of tight junction proteins like ZO-1, Occludin, and Claudin-11 (29). Furthermore, key Epimedium constituents Quercetin and Kaempferol boost Occludin expression in Sertoli cells, and Luteolin enhances the testicular microenvironment by activating the Nrf2 signaling pathway and upregulating the gap junction protein Connexin 43 (CX43) (30, 31). Additionally, in protecting spermatogenesis, total flavonoids, Icariin II and Icariside II reduce testicular oxidative stress damage and decrease germ cell apoptosis by enhancing antioxidant enzyme activity (24, 32, 33). These compounds show potential for mitigating blood-testis barrier disruption, enhancing testosterone production, and restoring spermatogenesis, providing pharmacological rationale for exploring Epimedium-derived therapies in Undescended Testis management.

Our findings identify Quercetin, Kaempferol, Icariin, Icariside II, Luteolin, and Yinyanghuo B as key active components of Epimedium. Quercetin modulates androgen receptor and TNF-α expression, suppresses oxidative stress and inflammation, and counteracts testicular dysfunction induced by lead acetate or aluminum oxide nanoparticles (34). Kaempferol enhances testicular weight, improves sperm quality (e.g., motility and morphology), and alleviates oxidative stress by reducing malondialdehyde while elevating glutathione levels, thereby potentially mitigating reproductive damage in male rats exposed to Echis ocellatus venom (35).

PPI network analysis highlights AKT1, TP53, IL6, TNF, ESR1, EGFR, PARP1, CASP3, and PGR as potential therapeutic targets. AKT1, critical for cell survival and apoptosis, regulates Sertoli cell development; its deficiency disrupts seminiferous tubule formation and accelerates germ cell death under stress (36). TP53, a tumor suppressor, inhibits DNMT1 and upregulates miR-199a/214 in testicular germ cell tumors (TGCT), impairing DNA methylation to exert anticancer effects (37). EGFR dysregulation (via mutation, amplification, or overexpression) correlates with elevated TGCT recurrence risk post-orchiectomy (38). These targets collectively underscore Epimedium’s multifaceted mechanisms in addressing Undescended Testis pathogenesis.

Functional enrichment analysis revealed that Epimedium targets biological processes linked to xenobiotic response, chemical stress adaptation, and oxidative stress mitigation, suggesting its role in modulating testicular recovery via environmental stress regulation. KEGG pathway analysis further identified key signaling cascades, including chemical carcinogenesis-receptor activation, endocrine resistance, AGE-RAGE, HHV-8 infection, and HIF-1 pathways. Epidemiological studies link prenatal endocrine-disrupting chemical exposure to Undescended Testis and impaired reproductive outcomes (39). The AGE-RAGE axis drives germ cell dysfunction by inducing oxidative stress and inflammation via AGE accumulation (40). Hypoxia and dihydrotestosterone deficiency in Undescended Testes disrupt Sertoli cell integrity, impairing spermatogenesis (41). Notably, HIF-1 pathway inhibition (e.g., by melatonin) alleviates hypoxia-induced Sertoli cell fibrosis (42). These findings highlight Epimedium’s multi-target potential in counteracting disease-related hypoxia, oxidative stress, and inflammatory pathways, supporting its therapeutic utility in Undescended Testis management.

Cells face constant exposure to DNA-damaging stressors, with unrepaired genomic lesions contributing to diseases like cancer (43). PARP1, the founding member of its family (44), plays critical roles in DNA repair by catalyzing poly (ADP-ribose) (PAR) polymer synthesis at damage sites through PARylation. This post-translational modification facilitates multiple repair pathways, including base excision repair and strand break resolution. Undescended Testis elevates testicular temperature, inducing oxidative stress and cellular damage linked to male infertility and germ cell tumors (45). Two-sample Mendelian randomization analysis results showed a significant causal relationship between elevated PARP1 levels and reduced risk of Undescended Testis. Heat exposure disrupts Sertoli cell function, compromises the blood-testis barrier, and triggers germ cell apoptosis (26, 45). In this study, we subjected the Sertoli cell line (TM4 cell line) to heat stress to simulate the pathological microenvironment of Undescended Testis. PCR experimental results showed that compared with the blank group, the levels of *Parp1* in TM4 cells subjected to heat stress were significantly increased, indicating that *Parp1* plays a key role in the repair of heat stress damage. Molecular docking demonstrated strong binding between PARP1 and Yinyanghuo B (binding energy -7.2 kcal/mol), indicating potential therapeutic modulation of PARP1 pathways.

Recent investigations have demonstrated that heat stress (HS)-induced tissue repair involves dynamic ubiquitination modulation, suggesting that the ubiquitination-dependent clearance of misfolded proteins may represent a pivotal mechanism for managing HS-related pathologies and Undescended Testis rehabilitation. Notably, our functional enrichment analysis failed to reveal significant associations with ubiquitination-related processes, potentially attributable to the predominant focus of the analyzed datasets on early-phase Undescended Testis pathogenesis mechanisms, with limited inclusion of contemporary findings regarding reversible testicular damage repair. Future work should expand cohorts, diversify data sources, and employ rigorous designs. Furthermore, while the *in vitro* and *in vivo* heat stress models used in this study simulate the injury mechanisms of Undescended Testis to some extent, they may not fully capture the complexity of human clinical Undescended Testis. While acute and chronic heat stress share fundamental pathophysiological mechanisms, Undescended Testis as a chronic heat stress condition involves multifaceted interactions of hormonal regulation, genetic predisposition, and environmental factors. The duration, severity of cumulative damage, and tissue adaptive responses cannot be fully replicated. Therefore, translating findings from acute heat stress models to clinical Undescended Testis requires additional validation through chronic heat stress studies and clinical investigations. It should be noted that this study primarily focused on predicting potential therapeutic targets for heat stress intervention through Mendelian randomization analysis, therefore employing only acute heat stress models for preliminary exploration. Secondly, our data were derived exclusively from European populations, limiting diversity in terms of ancestry, environment, and geographical region. Additionally, the Mendelian randomization analysis based on GWAS could be susceptible to confounding by other genetic or environmental factors, which were not fully accounted for in the analysis. Future work should expand cohorts, diversify data sources, and employ rigorous designs. In addition, combining animal model studies and multicenter clinical trials will help validate the findings and promote clinical applications.

The results of this study suggest that Epimedium and its active components may hold promise as adjunctive therapies for Undescended Testis, particularly Yinyanghuo B, which targets key proteins such as PARP1 to enhance testicular function. Future research should further investigate the clinical application of Epimedium in patients with Undescended Testis, with the aim of fostering the integration of Traditional Chinese Medicine and modern medical practices, thereby offering novel insights and therapeutic approaches for the treatment of male infertility.

## Data Availability

The original contributions presented in the study are included in the article/supplementary material. Further inquiries can be directed to the corresponding author.
